# Nutlin-3, the small-molecule inhibitor of MDM2, promotes senescence and radiosensitises laryngeal carcinoma cells harbouring wild-type p53

**DOI:** 10.1038/sj.bjc.6605739

**Published:** 2010-06-29

**Authors:** A K Arya, A El-Fert, T Devling, R M Eccles, M A Aslam, C P Rubbi, N Vlatković, J Fenwick, B H Lloyd, D R Sibson, T M Jones, M T Boyd

**Affiliations:** 1 Division of Surgery and Oncology, School of Cancer Studies, University of Liverpool, 5th Floor. UCD Building, Daulby Street, Liverpool L69 3GA, UK; 2 Department of Otolaryngology/Head and Neck Surgery, University Hospital Aintree, Liverpool, UK

**Keywords:** p53, MDM2, Nutlin-3, ionising radiation, laryngeal cancer, squamous cell carcinoma of the head and neck

## Abstract

**Background:**

Primary radiotherapy (RT) is a mainstay of treatment for laryngeal squamous cell carcinoma (LSCC). Although the cure rates for early (T1) vocal cord tumours are high, RT proves ineffective in up to a third of T3 carcinomas. Moreover, RT is associated with debilitating early- and late-treatment-related toxicity, thus finding means to de-escalate therapy, while retaining/augmenting therapeutic effectiveness, is highly desirable. p53 is a key mediator of radiation responses; we therefore investigated whether Nutlin-3, a small-molecule inhibitor of MDM2 (mouse double minute 2; an essential negative regulator of p53), might radiosensitise LSCC cells.

**Methods:**

We performed clonogenic assays to measure radiosensitivity in a panel of LSCC cell lines (for which we determined p53 mutational status) in the presence and absence of Nutlin-3.

**Results:**

LSCC cells harbouring wild-type p53 were significantly radiosensitised by Nutlin-3 (*P*<0.0001; log-rank scale), and displayed increased cell cycle arrest and significantly increased senescence (*P*<0.001) in the absence of increased apoptosis; thus, our data suggest that senescence may mediate this increased radiosensitivity.

**Conclusion:**

This is the first study showing Nutlin-3 as an effective radiosensitiser in LSCC cells that retain wild-type p53. The clinical application of Nutlin-3 might improve local recurrence rates or allow treatment de-escalation in these patients.

Squamous cell carcinoma of the head and neck (SCCHN) is the sixth commonest cancer in the world, with an estimated global incidence of 500 000 cases per year (Parkin *et al*, 2005). Despite recent advances in treatment strategies, the overall 5-year survival has not significantly improved over the last 20 years (http://www.cancerresearchuk.org.uk/cancerstats/index.htm) and currently stands at ∼60%. Radiotherapy (RT) remains one of the cornerstones of treatment for SCCHN, and a major objective in current research has been, and continues to be, to identify agents that may sensitise tumours to the effects of radiation, not only to improve efficacy but also to reduce treatment-associated toxicity. In the present study, we have examined the effects of one such agent, Nutlin-3.

The most commonly mutated gene in human cancers is the tumour suppressor gene p53 (*TP53*) and mutation of *TP53* has been implicated in ∼50% of all SCCHNs (Saunders *et al*, 1999; Poeta *et al*, 2007). The *TP53* gene encodes the protein p53, a transcriptional regulator that is activated in response to a wide range of cellular stresses, resulting either in a delay in cell cycle progression (providing an opportunity for DNA damage repair) and in some cases in senescence or in the initiation of programmed cell death (apoptosis) (for review see Vousden, 2006). As the p53 gene is pivotal in activating cellular responses to a wide range of stresses including DNA damage, it is not surprising that the ability of tumour cells to respond to chemo- and radiotherapy depends, at least in part, on the p53 pathway (Temam *et al*, 2000; Gudkov and Komarova, 2003; Soussi, 2003). The potential lethality resulting from activation of p53 requires reliable negative regulation, and two related genes, MDM2 (mouse double minute 2) and MDMX, have been proven to have critical roles in this process (Marine and Jochemsen, 2004; Toledo and Wahl, 2006). Of these two genes, the best understood is MDM2 (Iwakuma and Lozano, 2003). MDM2 negatively regulates p53, and recent studies have shown that the ability of MDM2 to act as a ubiquitin ligase targeting p53 for degradation by the proteasomes is essential for this (Itahana *et al*, 2007b). MDM2 is also a transcriptional target of p53, and thus an autoregulatory feedback loop regulates p53/MDM2 homoeostasis (Wu *et al*, 1993). In response to cellular stress such as DNA damage, post-translational modifications of p53 (and, although less well understood, of MDM2) prevent their interaction, resulting in increased levels of p53, which then activates target gene expression (Balint and Vousden, 2001; Meek and Knippschild, 2003; Bode and Dong, 2004). In normal cells, this ultimately leads to increased MDM2 expression, and in the absence of continued/further stress signaling, this returns p53 to pre-stress levels. MDM2 binds to p53 through a hydrophobic pocket on the MDM2 surface, and this provides a potential drug target (Kussie *et al*, 1996). Successful drug targeting of this site would present new opportunities for developing therapeutic regimens for cancers that retain wild-type p53. One drug that targets the p53-binding pocket of MDM2 and thus acts to inhibit p53/MDM2 interaction is Nutlin-3, developed by Roche (Nutley, NJ, USA) (Vassilev, 2004; Vassilev *et al*, 2004; Carvajal *et al*, 2005). Nutlin-3 is a small-molecule inhibitor that binds preferentially to the p53-binding pocket of MDM2, leading to stabilisation of p53 and activation of the p53 pathway. This has been shown to be effective in several models of cancer that harbour wild-type p53, including prostate, leukaemia and myeloma (Kojima *et al*, 2005; Stuhmer and Bargou, 2006; Lehmann *et al*, 2007). Moreover, Nutlin-3 has been shown to be an effective radiosensitiser in some forms of cancer (Cao *et al*, 2006; Lehmann *et al*, 2007).

The purpose of our study was to determine the effect of Nutlin-3 treatment on SCCHN cells. We focused on laryngeal squamous cell carcinomas (LSCC) for two reasons. First, cancers of the larynx are one of the most common sub-type of SCCHNs and second, to avoid potentially confounding effects resulting from the considerable biological heterogeneity manifested by SCCHNs from different anatomical sub-sites, which result in differences in their pathogenesis and prognosis (Berrino, 1998). In addition, as RT is a major treatment modality for SCCHNs including LSCC, we have examined whether Nutlin-3 alters radiosensitivity in LSCC cells. Our aim was to determine whether the use of a small-molecule inhibitor of MDM2 might provide a means to augment the treatment of patients with LSCC/SCCHNs, which would be based on the knowledge of the patient's p53 status.

## Methods

### Reagents

Mouse monoclonal antibodies against human MDM2 (Ab-1), p53 (Ab-6) and p21 (CDKN1A; Ab-1), were all purchased from Calbiochem (San Diego, CA, USA). The anti-actin (C-2, used as a total protein loading control) and the anti-p21 (anti-CDKN1A; F-5) antibodies were purchased from Santa Cruz Biotechnology (Santa Cruz, CA, USA). Secondary antibodies were anti-mouse HRP, used at a dilution of 1:2500 (from GE Healthcare, Little Chalfont, UK).

### Cell culture

The UM-SCC cell lines were kindly provided by Professor TE Carey, University of Michigan, USA (Krause *et al*, 1981; Carey *et al*, 1983; Frank *et al*, 1997). These were derived from patients known to have LSCC (Table 1). Cells lines were grown in Dulbecco's modified Eagle's media (Sigma, Poole, UK) containing 10% fetal bovine serum, 1% non-essential amino acids, 1% L-glutamine and 1% penicillin–streptomycin, and were incubated at 37°C in a humidified atmosphere containing 5% CO_2_.

### *TP53* gene mutation analysis

The PCR amplified exons 1–10 of the *TP53* gene were sequenced. The PCR primers were designed to include the entire exon-coding sequence and exon–intron junctions (Primer3 v0.4.0, Rozen and Skaletsky, 2000) as summarised in Supplementary Data Table 1. Genomic DNA (50 ng) was amplified in triplicate using HotStarTaq plus DNA polymerase (Qiagen, Crawley, UK), using an initial 95°C for 5 min, followed by 35 cycles of 94°C for 30 s, 61–65°C for 30 s, 72°C for 60 s and a 10 min at 72°C final extension. Residual primers and dNTPs were removed by exonuclease I and shrimp alkaline phosphatase (ExoSAP-IT, GE Healthcare, Little Chalfont, UK). DNA sequencing was performed using DYEnamic ET Dye Terminators (GE Healthcare). Sequencing reactions were purified by gel filtration (genClean 96-Well Dye Terminator Removal Kit; Genetix Limited, New Milton, UK) before analysis by capillary electrophoresis (Megabace 1000 DNA sequencing system; GE Healthcare). The resulting sequence was compared with the *Homo sapiens* chromosome 17 contig NT_010718.15, positions 7189581-7169068 bp, using Sequencher v 4.7 software (Gene Codes Corporation, Ann Arbor, MI, USA). Sequence variants were scored if they were present in both the sense and the antisense strand of all three replicates.

### Drug sensitivity evaluation

A total of 2 × 10^5^ cells were seeded into each well of a six-well plate and incubated for 24 h. After incubation, Nutlin-3 (a racemic mix of the active enantiomer, Nutlin-3a, and an inactive enantiomer, Nutlin-3b, obtained from Sigma) was dissolved in DMSO and diluted in complete media before adding to cells, which were then incubated as required. Cells were harvested by trypsinisation and then counted in triplicate by counting three independent samples using a Beckman Coulter Counter (Beckman Coulter (UK) Ltd., High Wycombe, UK (total cell number was counted not including any detached cells) or using an MTT assay, as indicated.

### Clonogenic assays

Cells harvested and counted as above were pre-incubated for at least 30 min with either Nutlin-3 or DMSO. A defined number of cells, as described in the legend to Figure 4 (determined empirically for each cell line), were irradiated with 0, 2, 4 or 6 Gy at room temperature using a ^137^Cs source delivering ∼6.25 Gy min^−1^ of *γ*-radiation. After irradiation, cells were seeded in triplicate into six-well plates and incubated for a period of 2–3 weeks to permit colony growth for counting (a colony was defined as a focus of ⩾50 cells, that is, more than five cell doublings). Plates were then washed with PBS and fixed and stained with a mixture of 6% (v/v) gluteraldehyde and 0.5% (w/v) crystal violet for at least 30 min. Plates were again washed and then left to air dry at room temperature overnight before counting colonies under a light microscope. Plating efficiencies were calculated and the survival data was fitted to the equation S(D)/S(0)=exp (−(*α*D+*β*D^2^)) to obtain survival parameters (Dale, 1985).

### Western blot analysis

Cells were typically seeded into 25 cm^2^ flasks and incubated overnight. The following day, cells were either treated with Nutlin-3, DMSO or left untreated. After incubation for a further 48 h, cells were harvested and proteins extracted as described previously (Brady *et al*, 2005). Typically, 50 *μ*g samples of total protein were separated by SDS–PAGE and transferred to Hybond ECL nitrocellulose membrane (GE Healthcare). Western blotting was performed as we have described previously (Brady *et al*, 2005) and signals were detected using Western Lightning chemiluminescence reagent (PerkinElmer, Cambridge, UK) and recorded and quantitated using a Kodak IS4000MM (Carestream Molecular Imaging, Woodbridge, CT, USA).

### Cell cycle and apoptosis assays

The DNA content and cell cycle distribution of cell populations was determined by flow cytometry. Cells were seeded as described above, and the next day drug or vehicle controls were added as required and cells were then cultured for 48 h. For some samples, after 30 min pre-incubation with either Nutlin-3 or DMSO, cells were exposed to 6 Gy irradiation and then incubated as above for 48 h. For DNA content analysis, cells were harvested and fixed in 70% ethanol before staining with 25 *μ*g ml^−1^ propidium iodide in a solution supplemented with 1 mg ml^−1^ RNase (Sigma) in PBS (pH 7.8) and then analysed on a FACSCalibur instrument (BD Biosciences, Oxford, UK). For apoptosis determination, cells were treated as above except that unfixed cells were analysed for Annexin-V surface expression using an Annexin-V-FLUOS staining kit (Roche) and then stained with propidium iodide according to the manufacturer's instructions.

### Senescence measurement

A senescence-associated *β*-galactosidase (SA-*β*-gal) assay (Dimri *et al*, 1995) was used to indicate the level of senescence in each cell line treated with Nutlin-3 or DMSO, with and without exposure to 6 Gy of irradiation. A total of 2 × 10^5^ cells were seeded into each well of a six-well plate, incubated for 24 h to permit attachment and then treated with either Nutlin-3 or an equal volume of DMSO for 48 h. Some samples were pre-incubated for 30 min with either Nutlin-3 or DMSO and then irradiated with 6 Gy of *γ*-radiation before seeding. After culturing for the indicated time, cells were washed with PBS and fixed for 15 min in a solution of 2% formaldehyde and 0.2% gluteraldehyde at room temperature. Cells were then washed twice in PBS and then incubated overnight at 37°C in 1 mg ml^−1^ X-gal (5-bromo-4-chloro-3-indolyl-*β*-D-galactopyranoside), 37 mM citric acid/127 mM di-sodium hydrogen phosphate (pH 6.0), 150 mM NaCl, 2 mM MgCl_2_, 5 mM potassium ferrocyanide and 5 mM potassium ferricyanide. The next day, cells were examined under a microscope for the development of staining indicative of senescence. Percentages of SA-*β*-gal-positive cells were determined by scoring 300 cells from each of three independent wells.

## Results

**Figure 1:**
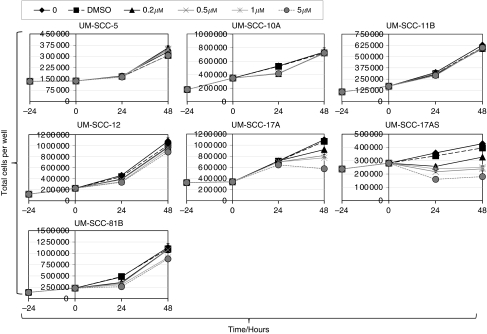
Nutlin-3 inhibits the proliferation of p53 wild-type laryngeal squamous cell carcinoma cells. Growth curves obtained for laryngeal squamous cell carcinoma cells in the presence and absence of Nutlin-3. A total of 1–2 × 10^5^ cells, depending on the cell line adherence and growth characteristics, were seeded in six-well plates and allowed to attach for 24 h after which they were either left untreated, treated with DMSO (the vehicle for Nutlin-3) or treated with Nutlin-3 at a range of concentrations, as indicated. Cells were harvested 24 and 48 h later and counted in a Beckman Coulter Counter. Error bars represent the s.e.m. from three independent wells. The results shown are from a typical experiment repeated on at least three occasions.

**Figure 2:**
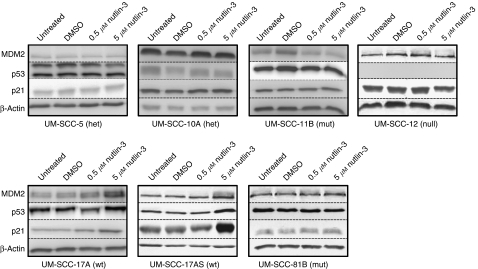
Nutlin-3 treatment induces p53 target gene expression in p53 wild-type laryngeal squamous cell carcinoma cells. Western blot analysis of laryngeal squamous cell carcinoma cells harvested 48 h after either no treatment, treatment with DMSO (vehicle control) or treatment with Nutlin-3 at 0.5 *μ*M or 5.0 *μ*M, as indicated. All lanes were loaded with 50 *μ*g of protein. Blots were probed with antibodies for p53, MDM2, p21 and *β*-actin (protein loading control) as indicated. Note the absence of any detectable p53 in UM-SCC-12 cells, known to be p53 null from DNA sequence analysis (Q104Stop, LOH). UM-SCC-5 shows two distinct bands of p53 protein, indicative of a well-documented polymorphism in codon 72, which was also confirmed by DNA sequence analysis (codon 72 is Pro/Arg, data not shown). The results shown are from a typical experiment repeated on at least three occasions.

**Figure 3:**
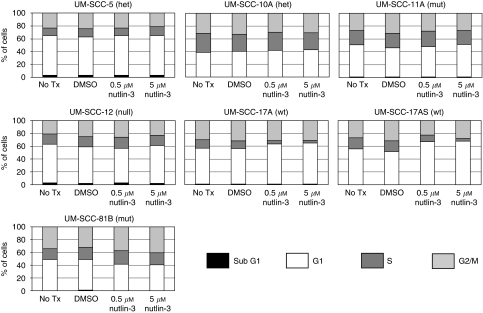
Nutlin-3 induces cell cycle arrest in p53 wild-type laryngeal squamous cell carcinoma cells. Histograms summarising data obtained by flow cytometry after propidium iodide staining (PI) of fixed laryngeal squamous cell carcinoma cells (also see Supplementary Data Figure 2). Cells were seeded and either left untreated (No Tx) or were treated for 48 h with DMSO, 0.5 *μ*M Nutlin-3 or 5 *μ*M Nutlin-3 as indicated. Cells were harvested, fixed in ethanol and stained with propidium iodide. The results shown are from a typical experiment repeated on at least three occasions.

**Figure 4:**
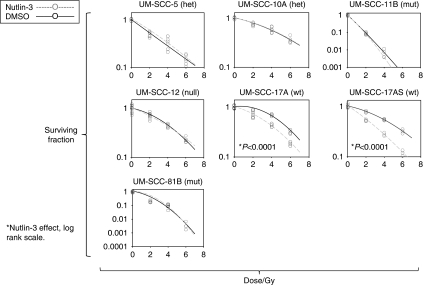
Nutlin-3 radiosensitises p53 wild-type laryngeal squamous cell carcinoma cells. Clonogenic assays were performed on laryngeal squamous cell carcinoma cells exposed to the indicated doses of γ-irradiation. Cells were pre-treated with either DMSO or 5 *μ*M Nutlin-3 for 30 min and then irradiated with 0, 2, 4 or 6 Gy as indicated. Cells were then re-seeded and allowed to form colonies for a period of 2–3 weeks. A colony was defined as containing at least 50 cells, equivalent to greater than five cell doublings. Colonies were fixed, stained and counted. The number of cells seeded varied according to the clonogenic characteristics of each cell line: 6400 for UM-SCC-5, 32 000 for UM-SCC-10A, 1600 for UM-SCC-11B, 400 for UM-SCC-12, 25 000 for UM-SCC-17AS, 6400 for UM-SCC-17A and 1600 for UM-SCC-81B. The quadratic equation S(D)/S(0)=exp(−(*α*D+*β*D^2^)) was used to obtain survival parameters, and all *r*^2^ values were >0.92. Note that cells expressing full-length mutant p53, in the absence of endogenous wild-type p53, were relatively radiosensitive compared with cells harbouring all other genotypes. The results shown are from a typical experiment repeated on at least three occasions.

Table 1 lists the cell lines used in this study, which were all derived from patients with primary squamous cell carcinoma of the larynx with the exception of UM-SCC-12 (recurrence after primary surgery). The confirmed *TP53* status of each cell line, analysed using DNA sequence analysis of the entire coding sequence including intron/exon boundaries, is also included in Table 1. In this group of seven cell lines, two are wild-type (UM-SCC-17A and UM-SCC-17AS), two harbour heterozygous mutations (UM-SCC-5 and UM-SCC-10A), two (UM-SCC-11B and UM-SCC-81B) harbour only mutant p53 (with loss of heterozygosity (LOH)) and one harbours a truncation mutation with LOH (p53 null; UM-SCC-12). The sensitivity of each cell line to varying concentrations of Nutlin-3 in the range from 0.2 to 5 *μ*M (range selected on the basis of both preliminary scoping experiments indicating that 10 *μ*M had no additional effect, which accords with other studies that have found that maximal effects of Nutlin are achieved *in vitro* at ⩽5 *μ*M (Vassilev *et al*, 2004; Tovar *et al*, 2006)) is depicted in Figure 1 and summarised in Table 1. Similar results were obtained using an MTT assay to monitor cells (see Supplementary Data Figure 1). Dose-dependent sensitivity to Nutlin-3 is displayed by both wild-type lines UM-SCC-17A and UM-SCC-17AS, whereas all of the mutant lines display essentially identical growth in the presence and absence of Nutlin-3. To investigate the effects of Nutlin-3 further, the steady-state protein levels of p53, MDM2 and p21 (CDKN1a) were measured by western blot analysis after 48 h of treatment with varying concentrations of the drug, as shown in Figure 2. As expected, p53 could not be detected in UM-SCC-12 cells, which possess only a truncated form of p53 (Q104Stop). UM-SCC-5 displayed two distinct bands of p53, suggestive of polymorphism. This was confirmed by sequencing, which showed that at codon72, this line harbours alleles encoding both proline and arginine (data not shown). As expected, only the two p53 wild-type cell lines (UM-SCC-17AS and UM-SCC-17A) displayed increased expression of p53, MDM2 and p21 after treatment with Nutlin-3. This accords with studies that have shown that the sensitivity of cells to Nutlin-3 is due to an increase of intracellular p53, with a concomitant rise in p21 expression (as well as MDM2) (Vassilev *et al*, 2004), although the role of p21 function has recently been called into question (Kan *et al*, 2007). Next, we determined the cell cycle profile of cells treated with Nutlin-3 by flow cytometry. Figure 3 summarises the results (also see Supplementary Data Figure 2) from a typical experiment, and shows that there is a reduction in the S-phase population and a concomitant increase in the G1 population in the wild-type cells with increasing doses of Nutlin-3. This is typical of cell cycle arrest, primarily in G1, and as Figure 2 suggested, this cell cycle arrest is likely to be caused by a p53-mediated increase in p21 expression in wild-type cells (Waldman *et al*, 1995; Vassilev *et al*, 2004). No such change is detectable in the cell cycle profile in any of the other cell lines (we have however noted evidence of a G2/M arrest (reduced G1 and increased G2/M population) in the p53-mutant line, UM-SCC-81B, an event that can be both p53 independent (Bernhard *et al*, 1995) and p53 dependent-mediated through 14-3-3*σ*; reviewed in Hermeking and Benzinger (2006). It has been shown previously that cells in G1 are approximately twice as radiosensitive as cells in S-phase (Pawlik and Keyomarsi, 2004), and therefore we considered the possibility that the cell cycle arrest that we observed in wild-type cells might also lead to increased radiosensitivity. It has been shown previously (Lehmann *et al*, 2007) that pre-treatment of prostate cancer cells with Nutlin-3 leads to increased radiosensitivity, and therefore we used a similar experimental regimen to investigate this in LSCC cells. We performed *in vitro* clonogenic assays for LSCC cells after short-term (30 min) pre-treatment with high-dose Nutlin-3 (5 *μ*M) or DMSO (vehicle control) followed by exposure to *γ*-radiation at 0, 2, 4 or 6 Gy, as illustrated in Figure 4. Pre-treatment with Nutlin-3 had a significant effect on the p53 wild-type cells, making them significantly more radiosensitive (comparison of SF2 values; *P*<0.0001, log-rank scale). As expected from our earlier data, Nutlin-3 treatment had no significant effect on any of the other cells. Perhaps, surprisingly, the survival curves obtained also show that cells with homozygous/LOH mutations of *TP53* are significantly (*P*<0.001) more radiosensitive than those with wild-type, heterozygous or null mutations, but we have not determined whether this is a causal relationship.

**Figure 5:**
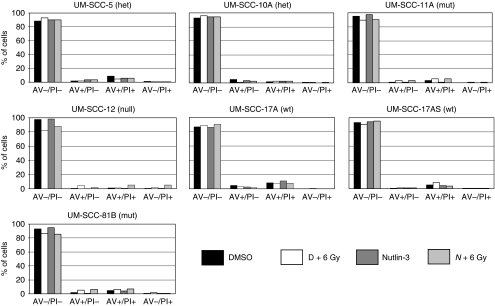
Analysis of apoptosis in laryngeal squamous cell carcinoma cells treated with ionising radiation and/or Nutlin-3. Summary of flow cytometric analysis of LSCC cells after either irradiation with 6 Gy or mock treatment. As indicated, cells were pre-treated for 30 min with either DMSO or 5 *μ*M Nutlin-3. After treatment, cells were re-seeded for 48 h before harvesting. Cells were then incubated with Annexin-V-FITC and propidium iodide to measure apoptosis and membrane integrity respectively. The log/log plot were divided into four quadrants as shown in Supplementary Data Figure 3, and the percentage of cells in each quadrant is presented in the histograms. Cells in the lower right quadrant stained with Annexin-V (AV+/PI−) are indicative of cells undergoing apoptosis and cells that have died are expected in the upper right quadrant staining for both Annexin-V and propidium iodide (AV+/PI+). Healthy/intact cells are negative for Annexin-V and propidium iodide (AV−/PI−). The results shown are from a typical experiment repeated on at least three occasions.

Having shown that Nutlin-3 pre-treatment of *TP53* wild-type cells results in a significant increase in radiosensitivity, we next set out to investigate the mechanism underlying this difference. Induction of p53 is well documented to induce apoptosis (Oren, 2003), and thus one simple explanation for the reduced survival after exposure to ionising radiation in the presence of Nutlin-3 is that this leads to increased p53-dependent apoptosis. As shown in Figure 3, flow cytometry did not detect any increase in the sub-G1 population of cells in response to Nutlin-3 treatment, and thus we conclude that this is unlikely to provide an explanation for this effect. Nevertheless, to further investigate this phenomenon, we used an alternative assay for apoptosis based on Annexin-V detection of surface-translocated phosphatidylserine (van Engeland *et al*, 1998). Figure 5 shows that Nutlin-3 has no detectable impact on Annexin-V positivity in response to 6 Gy of ionising radiation in any of the cells tested (also see Supplementary Data Figure 3). Irradiation does induce an increase in Annexin-V staining, indicative of apoptosis in the p53-mutant (presumed LOH) and null cells, but this was not affected by pre-treatment with Nutlin-3. Combining the data from Figures 3 and 5, we conclude that apoptosis is unlikely to mediate the increase in radiosensitivity that we have observed in p53 wild-type LSCC cells pre-treated with Nutlin-3.

**Figure 6:**
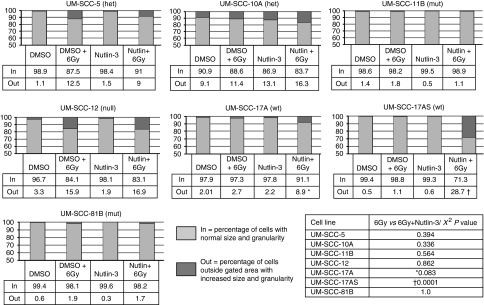
Laryngeal squamous cell carcinoma cells display increased complexity and/or size after exposure to ionising radiation and/or Nutlin-3. Flow cytometric analysis of the same samples shown in Figure 5. Forward *vs* side scatter plots were analysed to identify cells displaying unusual complexity and/or size (increased side scatter and/or forward scatter, respectively). Gates were applied to define the main population of cells and numbers of cells lying outside the gates were recorded. The data are presented in the table as the percentage of gated cells (denoted as ‘in’) and the percentage of more complex and/or larger cells lying outside the gates (denoted as ‘out’) shown as the darker bar. Cells lying outside the gates are larger and more granular, a phenotype typical of senescent cells. The results shown are from a typical experiment repeated on at least three occasions.

**Figure 7:**
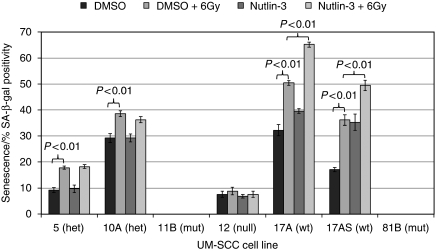
Increasing radiosensitivity in response to nutlin-3 treatment is associated with increased senescence. Senescence-associated *β*-galactosidase (SA-*β*-gal) assay of laryngeal squamous cell carcinoma cells after irradiation at 6 Gy after ∼30 min of pre-treatment with DMSO or 5 *μ*M Nutlin-3. Cells were treated and seeded for 48 h. A *β*-galactosidase assay was used at low pH (6.0), which stains senescent cells blue/green. A total of 300 cells were counted from three independent samples per condition, and the percentage of senescent cells as a fraction of the total was calculated. Error is expressed as the s.e.m. There is a clear increase in the number of senescent cells with Nutlin-3 treatment in *TP53* wild-type cells (UM-SCC-17AS and UM-SCC-17A), and also a further significant increase in senescence after irradiation, which is statistically significant (Student's *t*-test). Cells expressing full-length mutant p53 with LOH (UM-SCC-11B and UM-SCC-81B, the most radiosensitive) did not show any detectable senescence under any of the conditions examined. The results shown are from a typical experiment repeated on at least three occasions.

One possible explanation for the effect of Nutlin-3 that we have observed is that, upregulation of p53 might induce cellular senescence. Senescent cells undergo a number of morphological changes and one indication of this that has been reported is an increase in cell size and granularity, which can be detected as increased forward scatter and side scatter, respectively, by flow cytometry (Masterson and O’Dea, 2007). We therefore analysed the same samples shown in Figure 5, and as Figure 6 shows, we detected in one wild-type line, 17AS, a significant increase (*P*=0.0001) in the forward and side scatter of cells in response to exposure to 6 Gy of ionising radiation in cells pre-treated with Nutlin-3 (this was not significant in the other wild-type cell line, 17A; *P*=0.08). To simplify this analysis, we have scored cells according to whether they belong to the area defined by the majority cell population (that is, inside the plot frame denoted as ‘in’) or whether they have become so increased in size and/or complexity as to lie outside of the plot frame (denoted as ‘out’). It is striking that in the presence of Nutlin-3, both the p53 wild-type lines displayed a significant increase in size and/or granularity. We note that two other cell lines UM-SCC 5 and 12 also display a significant increase in response to 6 Gy of radiation, but this was not enhanced by the addition of Nutlin-3. Although increasing cell size and granularity are characteristics of senescent cells, we wanted to use a more direct and independent assay for senescence to determine whether these physical changes were accompanied by other characteristics of senescence. We therefore performed a SA-*β*-Gal assay (for review see Itahana *et al*, 2007a) on cells exposed to 6 Gy of ionising radiation with and without Nutlin-3 pre-treatment. Figure 7 shows that there is a significant increase (*P*<0.01) in the percentage of senescent p53 wild-type cells when these are pre-treated with Nutlin-3. We have also noted a significant increase in senescence after irradiation in the absence of Nutlin-3 (*P*<0.01) in these p53 wild-type cells.

Curiously, the highly radiosensitive cell lines UM-SCC-81B and 11B are the only ones not to display any detectable senescence in this assay under any of the conditions tested. Nevertheless, as far as the effect of Nutlin-3 is concerned, our observations support a possible role for senescence as a mechanism for radiosensitisation in p53 wild-type LSCC cells that warrants further investigation.

## Discussion

Improvements in cancer therapy can derive from the development of new drugs that target specific changes present in some cancer cells or indeed in their environment. In the former case, precise molecular information regarding each patient's cancer is required to select appropriate drugs. In the case of LSCC, although RT is effective in many cases, it is associated with significant treatment-related toxicity. Our study aims at addressing the therapeutic response (with potential implications for toxicity issues) by examining the use of a small molecule to increase radiosensitivity when targeted to individuals with disease that retains wild-type p53. We provide evidence that Nutlin-3 can indeed increase the sensitivity of p53 wild-type LSCC cells to RT and thus propose that this may provide an opportunity for de-escalating radiation dosage, without compromising cure rates, as part of a combination chemo–RT approach for a defined subset of LSCC patients. Conversely, the enhanced radiosensitivity mediated by Nutlin-3 might improve outcome at currently used radiation doses.

*Tp53* is the most frequently mutated gene in human cancer (Steele and Lane, 2005), and in SCCHN, mutation of p53 and, moreover, the type of p53 mutation (nonsense or disruptive missense *vs* non-disruptive missense) have been shown to be associated with reduced overall survival for surgically treated patients (Poeta *et al*, 2007). Nevertheless, although p53 mutation occurs frequently in patients with SCCHN (Olivier *et al*, 2002), approximately half of these patients retain potentially functional wild-type p53 (in a recent systematic review, we identified a mutation frequency for SCCHN of 50.7% in the United Kingdom (Tandon *et al*, 2010)). Our results from the present study suggest that this latter group might benefit from a combination chemo–RT approach that combines a p53 activator such as Nutlin-3, with RT, possibly permitting a de-escalation of the standard RT regimen and/or enhancing the therapeutic effectiveness of ionising radiation.

To avoid the potentially confounding effect of biological differences between SCCHN from different anatomical sub-sites, we have focused on LSCC in this study. However, it appears likely with respect to p53-targeted therapies that similar results may be obtained in most of the SCCHN sub-sites. Clearly, it will be necessary to determine the impact of potentially confounding factors on Nutlin-3 radiosensitisation. One obvious factor is likely to be infection with human papilloma virus (HPV). Human papilloma virus strains that promote cancer do so partly through inactivating p53, and cancers in some sub-sites, notably the oropharynx, display a relatively high incidence of HPV infection that might impact on the effectiveness of drug-induced radiosensitisation in this tissue (Hennessey *et al*, 2009). Nevertheless, the prospect of a personalised therapy with diminished treatment-related toxicity seems to be an attractive one for most other SCCHNs.

Our results in Figure 4 show that Nutlin-3 significantly (*P*<0.0001) reduces the ability of p53 wild-type LSCC cells to proliferate and form foci after exposure to ionising radiation. In studies of prostate cancer cells, p53 was shown to promote radiosensitivity and radiation-induced senescence (Lehmann *et al*, 2007). On the one hand, this clearly accords with the increased sensitivity and senescence that we have observed in LSCC cells harbouring wild-type p53 after treatment with Nutlin-3. Re-introduction of wild-type p53 in prostatic cancer cells has been shown to render them more sensitive to radiation, and also increases cellular senescence (Lehmann *et al*, 2007). These authors concluded that premature senescence, and not apoptosis, was the principal mode of death, which lead to failure of prostate cancer cells to proliferate after exposure to ionising radiation. From our data it seems that LSCC cells possess a similar pathway with similar consequences. On the other hand, we have observed that the most radiosensitive LSCC cells (UM-SCC-11B and 81B) also displayed no capacity for senescence under any of the conditions tested (as illustrated in Figures 4 and 7). This result seems to differ from the results obtained for prostate cancer cells and from our results using Nutlin-3. Such differences may be due to tissue-specific characteristics of prostatic and laryngeal cancer cells, but this observation requires further investigation to determine whether or not this reflects a causal association in LSCC cells. That these lines also exhibit p53 LOH calls for further functional analysis, but these issues lie outside the scope of this study. There is one additional observation that we have made that also relates to LOH. Not only does retention of one wild-type copy in the presence of a mutant p53 allele (heterozygous mutation) not render cells sensitive to the growth inhibitory effects of Nutlin-3 (Figure 1) but it also does not render the cells more radiosensitive (Figure 4). This contrasts with the ability of cells to undergo senescence, a property displayed by all cells in our study except those that exhibit p53 LOH (Figure 7). Whether this is due to p53-independent senescence induction or reflects a separation between p53 dominant-negative effects on two different phenotypes is unclear. Nevertheless, our data suggest that examination of these phenotypes may provide an experimental system to investigate such questions.

In this study, we have observed that increased radiosensitivity is linked with increased senescence. Senescence is usually defined as an irreversible process in which cell growth is arrested. Phenotypically, this is characterised in some cells by an increase in cytoplasmic volume and an accumulation of lysosomes, leading to increased granularity and upregulation of *β*-galactosidase expression (Dimri *et al*, 1995). The p53 gene has been shown to be capable of promoting a senescent phenotype in cells, but it remains unclear whether such p53-induced senescence is truly irreversible or rather represents a protracted, but ultimately reversible state (Efeyan *et al*, 2007; Ruiz *et al*, 2008). In one recent study, p53 was shown to be necessary for initiation of senescence in the presence of activated *Ras* in mouse embryo fibroblasts (MEFs) (Ruiz *et al*, 2008). However, it seems that p53 activity becomes downregulated in senescent cells and may not be required for maintenance of senescence. Perhaps of greater importance here, it was also found that maintaining growth arrest for long periods in MEFs did not result in a senescent phenotype in cells lacking an activated *Ras* oncogene. Oncogene activation is a *sine qua non* of cancer, and thus the LSCC cells that we have examined will certainly express at least one and likely several aberrant oncogenes, although as is typical of SCCHN (Ha and Califano, 2002), none of these cell lines harbour mutations in *KRAS* (our unpublished data). It seems likely therefore that the ‘senescence’ that we have detected in LSCC cells is not simply prolonged growth arrest, but is in fact irreversible genuine senescence.

In conclusion, our study shows that the use of p53-activating drugs, such as Nutlin-3, can provide an effective means of radiosensitisation in LSCC cells and likely in the broader context of SCCHN of known p53 status (specifically those with wild-type p53). Such an approach offers the prospect of a personalised combined chemo–RT intervention with reduced treatment-associated toxicity and/or improved outcome that could benefit approximately half of all patients with LSCC who are currently treated with RT.

## Figures and Tables

**Table 1:** Characteristics and *TP53* status of laryngeal squamous cell lines used in this study

**Cell line**	**TNM^a^**	**Grade^a^**	**Stage**	**Type of lesion**	**Primary location**	**Authentication**	**Primary reference**	**Reported p53 status^b^**	**p53 Status (this study)**	**Nutlin response**	**p53 Mutation effect**
UM-SCC-5	T2N1M0	PD	III	Primary	Supraglottis	D	Krause *et al* (1981)	Het: 157 gtc → ttc (transversion) V → F	Het: 157 gtc → ttc (transversion) V → F	R	DN
UM-SCC-10A	T3N0M0	M-WD	III	Primary	True vocal cord	D	Krause *et al* (1981)	Het: 245 ggc → tgc (transversion) G → C	Het: 245 ggc → tgc (transversion) G → C	R	DN
UM-SCC-11B	T2N2aM0		IV	Primary	Larynx		Carey *et al* (1983)	Mut: 242 tgc → tcc (transversion) C → S	Mut: 242 tgc → tcc (transversion) C → S	R	TA−ve (RGC)
UM-SCC-12	T2N1M0	MWD	III	Recurrence	Larynx		Carey *et al* (1983)	Het: 104 cag → tag (termination) Q → stop	Mut: 104 cag → tag (termination) Q → stop	R	TA−ve (RGC)
UM-SCC-17A	T1N0M0	MWD	I	Primary	Supraglottis	D	Carey *et al* (1983)	Wild type	Wild type^c^	S	
UM-SCC-17AS	T1N0M0	MWD		Primary	Supraglottis	D	Carey *et al* (1983)	Wild type	Wild type^c^	S	
UM-SCC-81B	T2N0M0	MWD	II	Primary	Larynx	D	Frank *et al* (1997)	Mut: 193 cat → cgt (transition) H → R	Mut: 193 cat → cgt (transition) H → R	R	DN?

Abbreviations: DN=dominant negative; DN?=questionable dominant negative; Het=heterozygous mutation, wild-type sequence is also present; mut=no wild-type sequence detected – loss of heterozygosity (LOH); M-WD=moderately to well differentiated; MWD=moderately well differentiated; PD=poorly differentiated; R=resistant; S=sensitive; TA−ve=compromised transcriptional activation function (IARC database); TNM=Tumour node metastasis; WD=well differentiated.

a TNM classification and staging is according to the American Joint Committee on cancer of the larynx.

b D indicates that the tumor cell lines were originally compared with microsatellite polymorphisms from normal tissue or cells from the same individual, as described in Frank *et al* (1997).

c 17A and 17AS are morphologically distinct and 17A grows more slowly.
