# Supplementary material for: Nutlin-3, the small-molecule inhibitor of MDM2, promotes senescence and radiosensitises laryngeal carcinoma cells harbouring wild-type p53
Source: Br J Cancer. 2010 Jun 29;103(2):186–95. doi: 10.1038/sj.bjc.6605739 (PMC2906734; doi:10.1038/sj.bjc.6605739)
Supplement: Supplementary Information [file 6605739x1.ppt]

## Slide 1
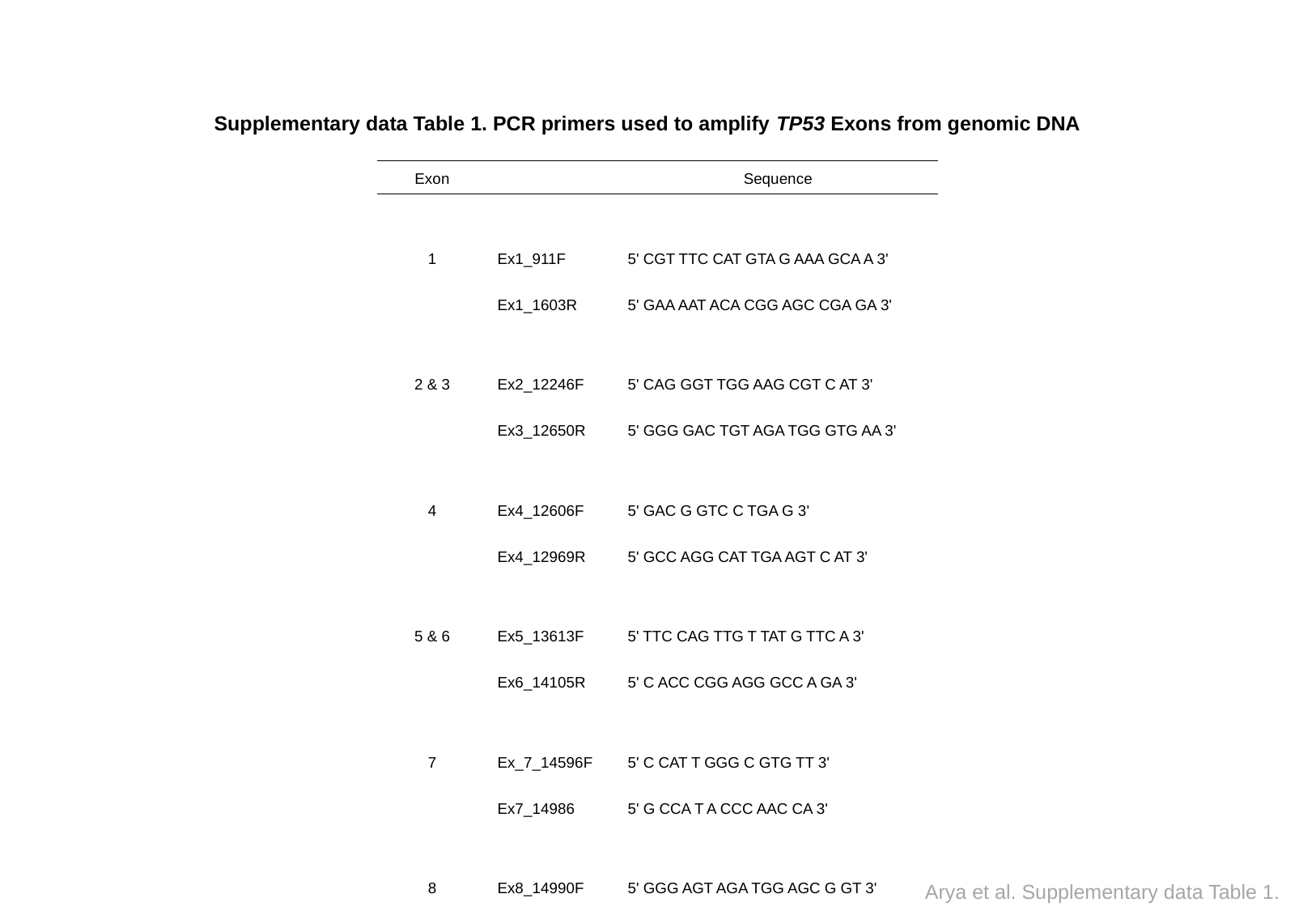

Supplementary data Table 1. PCR primers used to amplify TP53 Exons from genomic DNA
| Exon | | Sequence |
| --- | --- | --- |
| | | |
| 1 | Ex1\_911F | 5' CGT TTC CAT GTA G AAA GCA A 3' |
| | Ex1\_1603R | 5' GAA AAT ACA CGG AGC CGA GA 3' |
| | | |
| 2 & 3 | Ex2\_12246F | 5' CAG GGT TGG AAG CGT C AT 3' |
| | Ex3\_12650R | 5' GGG GAC TGT AGA TGG GTG AA 3' |
| | | |
| 4 | Ex4\_12606F | 5' GAC G GTC C TGA G 3' |
| | Ex4\_12969R | 5' GCC AGG CAT TGA AGT C AT 3' |
| | | |
| 5 & 6 | Ex5\_13613F | 5' TTC CAG TTG T TAT G TTC A 3' |
| | Ex6\_14105R | 5' C ACC CGG AGG GCC A GA 3' |
| | | |
| 7 | Ex\_7\_14596F | 5' C CAT T GGG C GTG TT 3' |
| | Ex7\_14986 | 5' G CCA T A CCC AAC CA 3' |
| | | |
| 8 | Ex8\_14990F | 5' GGG AGT AGA TGG AGC G GT 3' |
| | Ex8\_15237R | 5' G T TGT C G TGC TT 3' |
| | | |
| 9 | Ex9\_15142F | 5' GCG CAC AGA GGA AGA GAA TC 3' |
| | Ex9\_15511R | 5' TGT T TGA GGC ATC A GC 3' |
| | | |
| 10 | Ex10\_18125F | 5' CAA TTG TAA T GAA CCA T TTT AAC 3' |
| | Ex10\_18521R | 5' C GGG TTT GGA TGT T GT 3' |
Arya et al. Supplementary data Table 1.

## Slide 2
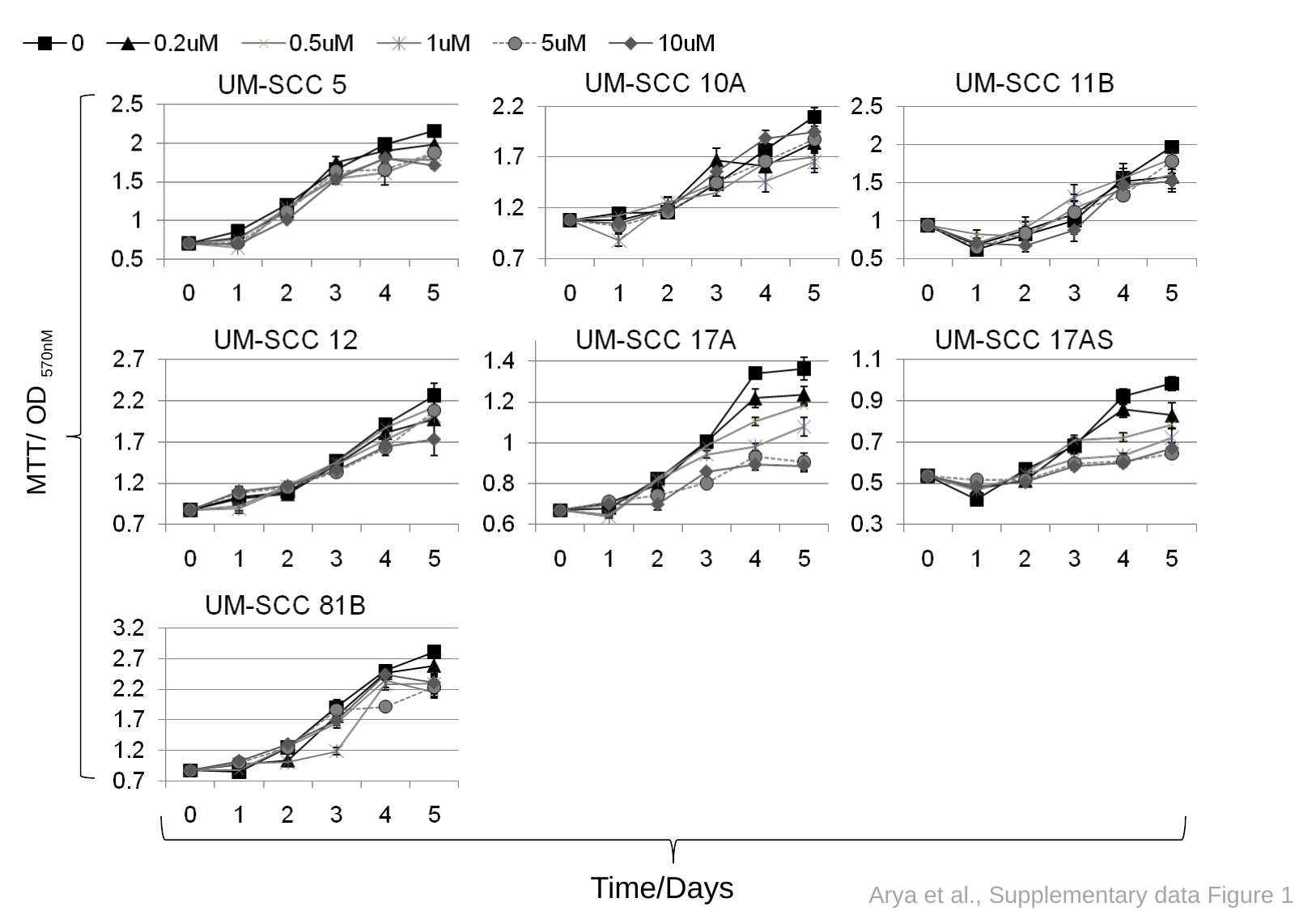

MTT/ OD 570nM
Time/Days
Arya et al., Supplementary data Figure 1

## Slide 3
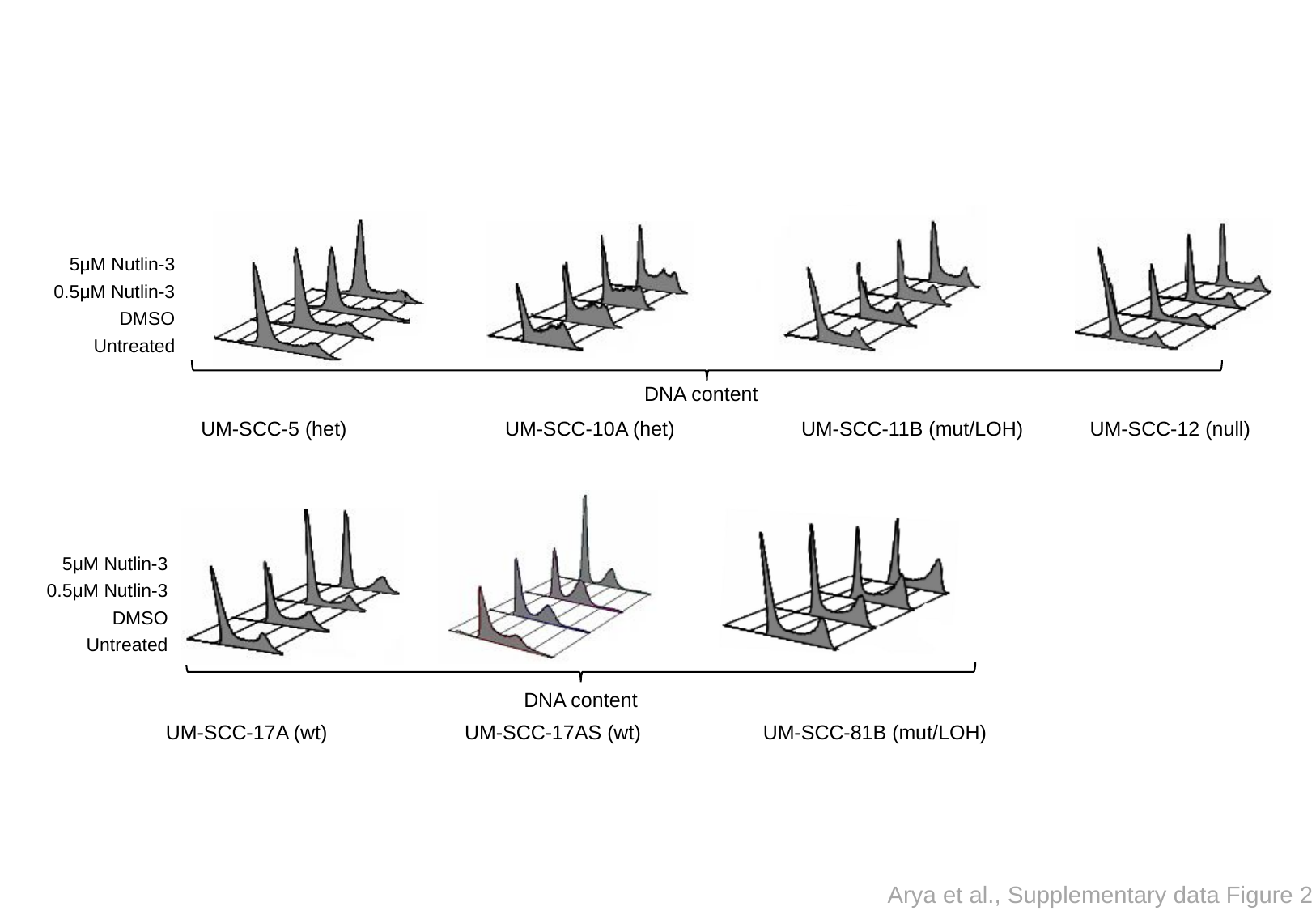

5μM Nutlin-3
0.5μM Nutlin-3
DMSO
Untreated
DNA content
UM-SCC-5 (het)
UM-SCC-10A (het)
UM-SCC-11B (mut/LOH)
UM-SCC-12 (null)
5μM Nutlin-3
0.5μM Nutlin-3
DMSO
Untreated
DNA content
UM-SCC-17A (wt)
UM-SCC-17AS (wt)
UM-SCC-81B (mut/LOH)
Arya et al., Supplementary data Figure 2

## Slide 4
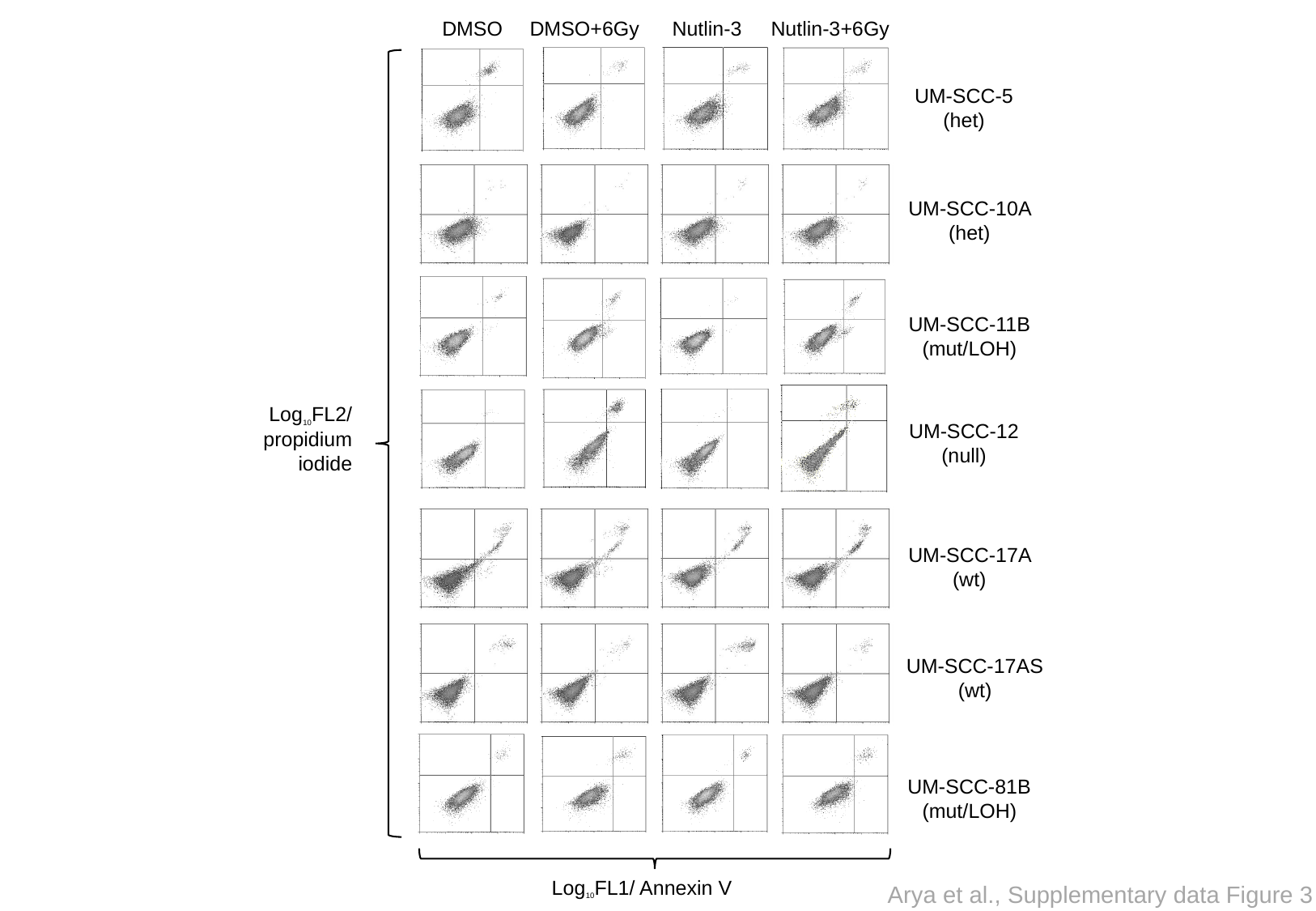

DMSO
DMSO+6Gy
Nutlin-3
Nutlin-3+6Gy
UM-SCC-5 (het)
UM-SCC-10A (het)
UM-SCC-11B (mut/LOH)
Log10FL2/ propidium iodide
UM-SCC-12 (null)
UM-SCC-17A (wt)
UM-SCC-17AS (wt)
UM-SCC-81B (mut/LOH)
Log10FL1/ Annexin V
Arya et al., Supplementary data Figure 3
